# Coexistence of Pancytopenia and Myositis After Developing COVID-19

**DOI:** 10.7759/cureus.26978

**Published:** 2022-07-18

**Authors:** Tatsuhiko Okayasu, Ryuichi Ohta, Mari Igarashi, Yasuo Kurita, Miho Hayakawa, Chiaki Sano

**Affiliations:** 1 Family Medicine, International University of Health and Welfare, Tokyo, JPN; 2 Communiy Care, Unnan City Hospital, Unnan, Shimane, JPN; 3 Education, International University of Health and Welfare, Tokyo, JPN; 4 Cardiology, International University of Health and Welfare, Tokyo, JPN; 5 Community Medicine Management, Shimane University Faculty of Medicine, Izumo, JPN

**Keywords:** community hospitals, japan, rural, autoantibody myositis, idiopathic neutropenia, covid-19

## Abstract

COVID-19 causes not only acute but also subacute medical conditions during the clinical course. COVID-19 causes severe inflammatory conditions; therefore, patients may develop long-term complications. Among patients with acute COVID-19, some patients can experience persistent symptoms, such as fatigue, joint pain, and smell and taste abnormalities, known as the long COVID-19 syndrome. The symptoms can be severe and require continuous medical care. Patients with severe clinical courses of COVID-19 may have critical symptoms again after the cure of the acute infections, especially among older patients. We encountered a case of neutropenia and myositis one month after contracting COVID-19. An 89-year-old man presented to our hospital with acute-onset systemic muscle pain and difficulty in movement and speaking. The patient had neutropenia and myositis with an extremely high level of immunoglobulin G caused by COVID-19. A granulocyte colony-stimulating factor could be effective for treating neutropenia. Besides, prednisolone was effective for treating myositis. In community hospitals, after developing COVID-19, appropriate history taking and physical examination should be performed in older patients with ambiguous symptoms, as they might have critical medical conditions such as neutropenia and myositis. The appropriate diagnosis and treatments of older patients with the complications of COVID-19 should be performed.

## Introduction

Coronavirus disease 2019 (COVID-19) is a respiratory infection induced by severe acute respiratory syndrome coronavirus‐2 (SARS‐CoV‐2), affecting human lives since 2019. COVID-19 causes severe respiratory infections with an average mortality rate of 3%-4% [1]. There are no definitive treatments for the virus, and vaccination is the only measure to prevent the spread of the infection [2,3]. COVID-19 also causes various systemic symptoms by triggering inflammatory conditions with high concentrations of interleukin 6 and tumor necrosis factors in the blood [4]. Concrete symptoms include fatigue, muscle pain, headache, and joint pains [5]. Moreover, the infection can cause myositis and hematological abnormalities during the acute infection phase; therefore, intensive monitoring of the infection is required [5].

COVID-19 causes not only acute but also subacute medical conditions during the clinical course. COVID-19 also causes severe inflammatory conditions; therefore, patients may develop long-term complications [6]. Among patients with acute COVID-19, some can experience continuous symptoms such as fatigue, joint pain, and smell and taste abnormalities, known as long COVID-19 syndrome [6,7]. Their symptoms can be severe and require continuous medical care [6,7]. COVID-19 patients with severe acute symptoms may develop critical symptoms again after the cure, which could be fatal, especially among older patients [6,8].

This time, we encountered an older patient with post-COVID-19 infections with complaints of appetite loss and systemic muscle pain who were transferred to the emergency room of our rural hospital. The patient developed myositis and pancytopenia, following acute COVID-19 infection. Myositis and pancytopenia rarely coexist in patients as COVID-19 complications. Only a few case reports on patients with the same conditions, especially among older patients, have been published [9,10]. We describe this rare case and discuss how COVID-19 causes these complications and the appropriate treatment for these patients in rural hospitals.

## Case presentation

An 89-year-old man presented to our hospital with acute-onset generalized muscle pain and difficulty in standing and speaking. His past medical histories were hypertension, angina pectoris, dementia, clavicle fracture, and lumbar vertebral compression fracture, and was admitted to our hospital after contracting COVID-19 one month before the present visit. He was under treatment with bisoprolol fumarate, tolvaptan, azosemide, aspirin, furosemide, and rupatadine fumarate.

Upon arrival, his vital signs were as follows: temperature, 37.7°C; pulse rate, 81 beats/min; respiration, 16 breaths/min; and blood pressure, 132/73 mmHg. Physical examination revealed tenderness of the proximal muscles of the extremities and bilateral pedal edema. He had stable breathing and an absence of chills, shivering, or cyanosis. The blood tests revealed pancytopenia (white blood cells: 900/µL, neutrophils: 1.2%, red blood cells: 282 × 104/µL, hemoglobin: 9.8 g/dL, and platelet count: 11.8 × 104/µL) and elevated creatinine kinase (2581 U/L) (Table 1).

**Table 1:** Patients’ initial laboratory data PT, prothrombin time; INR, international normalized ratio; APTT, activated partial thromboplastin time; eGFR, estimated glomerular filtration rate; CK, creatine kinase; CRP, C-reactive protein; TSH, thyroid-stimulating hormone; Ig, immunoglobulin; HCV, hepatitis C virus; SARS-CoV-2, severe acute respiratory syndrome coronavirus 2; HIV, human immunodeficiency virus; HBs, hepatitis B surface antigen; HBc, hepatitis B core antigen; C3, complement component3; C4, complement component4; KL-6, Krebs von den Lungen-6; MPO-ANCA, myeloperoxidase-antineutrophil cytoplasmic antibodies; anti-SSA/Ro autoantibodies, anti-Sjogren’s syndrome type A autoantibodies; anti-SSB/La autoantibodies, anti-Sjogren syndrome antigen type B autoantibodies; CCP antibodies, cyclic citrullinated peptide antibodies

Marker	Level	Reference
White blood cells	0.9	3.5–9.1 × 10^3^/μL
Neutrophils	1.2	44.0–72.0%
Lymphocytes	86.9	18.0–59.0%
Monocytes	6.0	0.0–12.0%
Eosinophils	0.0	0.0–10.0%
Basophils	0.0	0.0–3.0%
Red blood cells	2.82	3.76–5.50 × 10^6^/μL
Reticulocytes (%)	14.3	/μL (%)
Hemoglobin	9.8	11.3–15.2 g/dL
Hematocrit	29.0	33.4–44.9%
Mean corpuscular volume	102.8	79.0–100.0 fl
Platelets	11.8	13.0–36.9 × 10^4^/μL
PT-INR	0.98	
APTT	27.5	25–40 seconds
Total protein	8.2	6.5–8.3 g/dL
Albumin	3.6	3.8–5.3 g/dL
Total bilirubin	1.6	0.2–1.2 mg/dL
Aspartate aminotransferase	66	8–38 IU/L
Alanine aminotransferase	18	4–43 IU/L
Alkaline phosphatase	85	106–322 U/L
γ-Glutamyl transpeptidase	19	<48 IU/L
Lactate dehydrogenase	232	121–245 U/L
Blood urea nitrogen	32.2	8–20 mg/dL
Creatinine	2.13	0.40–1.10 mg/dL
eGFR	23.3	> 60.0 mL/min/L
Serum Na	140	135–150 mEq/L
Serum K	4.1	3.5–5.3 mEq/L
Serum Cl	102	98–110 mEq/L
Ferritin	330.8	14.4–303.7 ng/mL
CK	2581	56–244 U/L
CRP	11.79	<0.30 mg/dL
Procalcitonin	0.48	0–0.05 ng/mL
TSH	3.07	0.35–4.94 μIU/mL
Free T4	0.6	0.70–1.48 ng/dL
Vitamin B12	362	187–883 pg/mL
Folic acid	9.8	3.1–20.5 ng/mL
IgG	111	870–1,700 mg/dL
SARS-CoV-2 antigen	Negative	
SARS-CoV-2 IgG	＞40,000.0	<49.9 AU/mL
Antinuclear antibody	＜40	<40
Homogeneous	0	<40
Speckled	0	<40
Nucleolar	0	<40
Peripheral	0	<40
Discrete	0	<40
Cytoplasm	0	<40
C3	126	86–160 mg/dL
C4	31	17–45 mg/mL
KL-6	332	105.3–401.2 U/mL
MPO-ANCA	<1.0	<3.5 U/mL
Anti-SSA/Ro autoantibodies	<1.0	<10 U/mL
Anti-SSB/La autoantibodies	<1.0	<10 U/mL
CCP Antibodies	<0.6	<5 U/mL
Cardiolipin antibodies	<4.0	<12.3 U/mL
IgG4	111	11–121
Urine test		
Leukocyte	Negative	
Nitrite	Negative	
Protein	2+	
Glucose	Negative	
Urobilinogen	1+	
Bilirubin	Negative	
Ketone	Negative	
Blood	3+	
pH	8.0	
Specific gravity	1.016	
Bacteria	2+	

**Figure 1:**
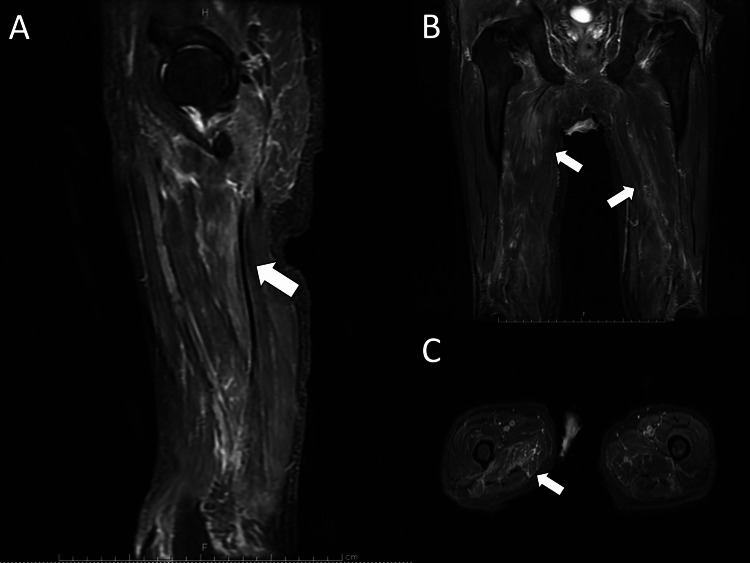
Short TI inversion recovery images of thigh muscle groups (A: Sagittal, B: Coronal, C: Transverse) showing muscle inflammation (white arrow)

Short Tau inversion recovery (STIR) imaging of the thighs showed irregular high-intensity areas in both adductor muscle groups, suggesting necrotizing fasciitis (Figure 1).

A chest computed tomography scan was performed and did not show obvious interstitial pneumonia or lymphadenopathy suspicious for lymphoid species. Antibody tests showed the absence of collagen-related antigen antibodies but demonstrated high levels of anti-coronavirus immunoglobulin G (40,000 U/mL) (Table 1). Based on these findings, febrile neutropenia due to COVID-19 and COVID-19 antibody-associated myositis was diagnosed.

**Figure 2:**
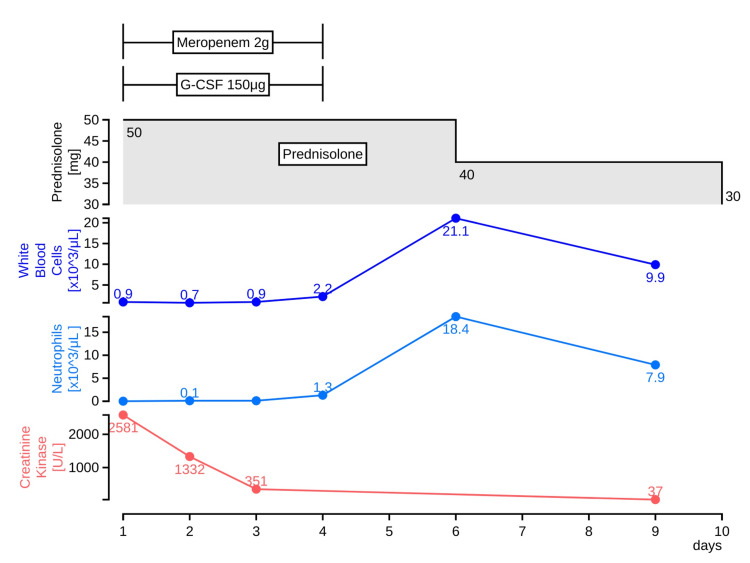
The clinical course of the case G-CSF:  a granulocyte-colony stimulating factor

The patient did not exhibit significant elevation of ferritin levels (330.8 ng/mL), and the inflammation affecting the bone marrow was considered mild. On the day of admission, a granulocyte-colony stimulating factor (G-CSF) of 75 μg was initiated as a treatment for neutropenia (neutrophil count of <100/μL). Since febrile neutropenia was observed until the fourth day of admission, the dose was increased to 150 μg; the febrile neutropenia was suspected to be caused by multidrug-resistant *Pseudomonas aeruginosa*. For COVID-19 antibody-associated myositis, prednisolone 50 mg was administered orally for five days (Figure 2).

On the fourth day of hospitalization, *P. aeruginosa* was reported in the urine culture submitted at the visit; on the fifth day of hospitalization, the white blood cell count increased to 2,200/µL, while the neutrophil count increased to 1,300/µL. Therefore, G-CSF and meropenem were discontinued, and prednisolone for myositis was tapered off on the sixth day of admission. The patient was discharged on the 10th day of hospitalization and underwent outpatient follow-up.

## Discussion

In this case, the patient presented with myositis and febrile neutropenia following acute COVID-19. This case suggests that neutropenia and myositis could be caused when the inflammation due to COVID-19 is severe and that G-CSF and prednisolone treatment may be effective against febrile neutropenia myositis following COVID-19 infection, although no clear literature evidence exists to support this finding.

Systemic inflammation and consequent autoantibody production after COVID-19 may affect the bone marrow and muscle, causing febrile neutropenia and autoantibody myositis, and should be considered in future cases of COVID-19. In our case report, the patient had no major symptoms at the time of COVID-19 but developed severe long-term complications. The complications of COVID-19 develop regardless of their severity, and the common symptoms include fatigue, dyspnea, anxiety, and depression [11,12]. Abnormalities in multiple organs, such as the lungs, renal tubules, heart, and liver, may also occur. In cases reported to date, neutropenia, and autoantibody myositis developed after contracting COVID-19 [13,14]. However, the pathophysiology of these cases remains unclear. According to previous studies, autoimmunity is temporarily activated by an abnormal increase in antibodies to COVID-19 and its cross-reactivity [13,14]. Most of the current case reports described neutropenia and myositis after developing COVID-19 [13-15]. However, none of the existing reports described the occurrence of only one of these conditions in COVID-19 patients. The present case suggests that the activation of autoimmunity by COVID-19 may spread to multiple organs rather than to a single organ, and further investigation is needed.

Further academic research regarding the use of G-CSF for febrile neutropenia after developing COVID-19 and prednisolone for antibody-associated myositis is warranted. In our patient, the continued use of G-CSF for febrile neutropenia and antimicrobial therapy enabled symptom control without abrupt changes. Previous literature suggests that G-CSF may be effective in the treatment of febrile neutropenia after contracting COVID-19; the post-COVID-19 hyperinflammatory state begins several weeks after onset, and there is no sufficient literature reporting the duration of this state [15,16]. There is a lack of evidence reporting the duration of the G-CSF administration. The mechanism is different from that of an immunocompromised host with febrile neutropenia after developing malignancy and undergoing chemotherapy, and the condition may be less likely to be severe. However, it is important to continue monitoring the neutrophil count and treating patients in the same manner as those with febrile neutropenia. With regard to COVID-19 antibody-associated myositis, a relationship between COVID-19 infection and autoimmune disease has been reported, although the mechanism of this relationship remains unclear [15,16]. In our case, the patient was suspected of having an autoimmune disease that could cause myositis and underwent blood tests, which showed the absence of antibodies except for an abnormal SARS-CoV-2 immunoglobulin G (IgG) level elevation. Based on the clinical course of this case, myositis was considered to have been caused by the release of autoantibodies during COVID-19. Although the appropriate treatment for this case was not clearly indicated in the previous literature, there were scattered reports of cases treated with prednisolone [15,16]. In the present case, the use of 50 mg prednisolone reduced the pain, which could be tapered off and discontinued from the 10th day of the disease. In conclusion, antibody-associated myositis is due to the transient elicitation of autoinflammation rather than the general spectrum of autoimmune diseases and may be in remission after short-term steroid use.

The current spread of COVID-19 may lead to an increase in similar cases. In community hospitals in Japan, many patients are older, and their symptoms are often vague [17,18]. Older individuals also have a variety of medical treatment behaviors and may not be able to appropriately self-manage their illnesses or use medical resources [17]. The spread and fear of COVID-19 may lead to an increase in the number of older patients with a variety of symptoms [19]. Clinicians working in the community should take a detailed history and physical examination; if there is a history of COVID-19, high inflammation caused by COVID-19 and inflammatory conditions caused by autoantibodies should be considered as differential diagnoses, and appropriate diagnosis and treatment should be provided [20].

## Conclusions

This patient presented with myositis and febrile neutropenia induced following acute COVID-19. This case suggests that neutropenia and myositis could be caused by severe infection of COVID-19 and high titer of IgG against COVID-19. G-CSF and prednisolone therapy may be effective in treating febrile neutropenia and myositis that develops after COVID-19 infection. In community hospitals, it is necessary to take an appropriate history and physical examination of elderly patients with ambiguous symptoms after COVID-19 and to diagnose various complications by acute inflammation caused by COVID-19.
